# Supplementary material for: Multi-benthic size approach to unveil different environmental conditions in a Mediterranean harbor area (Ancona, Adriatic Sea, Italy)
Source: PeerJ. 2023 Jun 28;11:e15541. doi: 10.7717/peerj.15541 (PMC10314744; doi:10.7717/peerj.15541)
Supplement: Supplemental Information 1 — The raw data shows environmental parameters and benthic fauna measurements, as well as statistical analysis results. Phaeo, Phaeopigment concentration; CPE, chloroplast pigment equivalents; PRT, Protein concentration; CHO, Carbohydrate concentration; LIP, Lipid concentration. Mean values ± standard deviation are reported. [file peerj-11-15541-s001.docx]

**Table S1. Multicollinearities among variables.** Within brackets the Spearman's Rho value. Two variables have been considered as collinear if Rho ≥ |0.8|. In bold the variables used for the PCA to characterize the harbor area environment. In italics the two variables that have been included (with the others) in the Varimax process of variable reduction (see main text for explanation).

|  | Variable | Collinear to (Spearman's Rho) |
| --- | --- | --- |
|  |  |  |
| Biotics | Chla | FEO (0.94); CPE (0.94) |
|  | Feo | Chla (0.94); CPE (0.99) |
|  | **CPE** | FEO (0.99); Chla (0.94); NAPH (0.81) |
|  | PRT | LIP (0.79); BPC (0.98) |
|  | ***CHO*** | Cu (0.83); NAPH (0.80); ANT_PHE (-0.87) |
|  | LIP | BPC (0.81); PRT (0.79) |
|  | **BPC** | PRT (0.98); LIP (0.81) |
|  |  |  |
| Environmental | S | Depth (0.9); BAP (-0.86) |
|  | Depth | S (0.9) |
|  | **%mud** | Hg (0.85) |
|  |  |  |
| Metals | As | Cd (0.89); Cr (0.83); Pb (0.9); Zn (0.84) |
|  | Cd | As (0.89); Pb (0.96); Zn (0.96) |
|  | **Cr** | As (0.83) |
|  | Cu | CHO (0.83) |
|  | Fe | FLU (-0.78) |
|  | Hg | %mud (0.85); FLU (-0.82) |
|  | **Mn** | - |
|  | Pb | As (0.9); Cd (0.96); Zn (0.98) |
|  | **Zn** | As (0.84); Cd (0.96); Pb (0.98) |
|  | **Ni_V** | - |
|  |  |  |
| Organic compounds | ***NAPH*** | 1-MeNAP (0.9); 2-MeNAP (0.78); CPE (0.81); CHO (0.80) |
|  | 1-MeNAP | NAPH (0.9); 2-MeNAP (0.96); BAP (0.93); ANT_PHE (-0.84) |
|  | 2-MeNAP | NAPH (0.9); 1-MeNAP (0.96); BAP (0.94); ANT_PHE (-0.83) |
|  | **FLU** | Fe (-0.78); Hg (-0.82) |
|  | BBF | BAP (0.79) |
|  | BKF | BPER (0.78) |
|  | **BAP** | 1-MeNAP (0.93); 2-MeNAP (0.94); BBF (0.79); S (-0.86) |
|  | **BPER** | BKF (0.78) |
|  | **ANT_PHE** | 1-MeNAP (0.84); 2-MeNAP (0.83); CHO(-0.87) |
|  | **FLT_PYR** | - |

**Table S2.** **Location of the five sampling stations, environmental variables measured during the sampling, grain size,** **biochemical composition, ~~and microbial component~~ of the sediment samples.** Chl-*a* = Chlorophyl-*a* concentration; BPC = Biopolymeric organic carbon; PRT/CHO = Protein to carbohydrate ratio; ~~TPN = Total Prokaryotic Number~~. Mean values ± standard deviation are reported.

| Sampling station | | **MAN** | **PORT** | **DS** | **LR** | **API** |
| --- | --- | --- | --- | --- | --- | --- |
| Latitude N | | 43°36.793 | 43°37.020 | 43°37.130 | 43°37.309 | 43°40.297 |
| Longitude E | | 13°30.174 | 13°30.317 | 13°29.482 | 13°29.400 | 13°24.344 |
| Water temperature (°C) | | 10.4 | 10.2 | 10.2 | 10.1 | 10.7 |
| Salinity (PSU) | | 32.0 | 32.5 | 36.7 | 31.3 | 37.6 |
| Depth (m) | | 3.1 | 6.2 | 11.0 | 5.0 | 14.0 |
| Grain size | Sand% | 2 | 20 | 84 | 84 | 79 |
|  | Silt/mud% | 98 | 80 | 16 | 16 | 21 |
| Chl-*a* (μg g^-1^) | | 13.9 ± 5.2 | 2.3 ± 0.4 | 17.0 ± 4.6 | 8.1 ± 1.2 | 2.9 ± 0.6 |
| BPC (mgC g^-1^) | | 10.2 ± 1.8 | 5.7 ± 1.5 | 3.7 ± 0.3 | 4.5 ± 0.5 | 2.3 ± 0.5 |
| PRT/CHO | | 5.5 ± 1.5 | 40 ± 16.1 | 5.2 ± 1.4 | 4.2 ± 1.5 | 12.9 ± 2.7 |
| ~~TPN (no. of cells mL~~^~~-1~~^~~)~~ | | ~~1.8E+06 ± 4.0E+05~~ | ~~8.4E+05 ± 7.4E+05~~ | ~~1.0E+06 ± 6.1E+05~~ | ~~3.8E+05 ± 3.7E+05~~ | ~~1.3E+06 ± 1.2E+05~~ |

**Table S3.** **Biochemical composition of the sediment samples**. Phaeo = Phaeopigment concentration; CPE = chloroplast pigment equivalents; PRT = Protein concentration; CHO = Carbohydrate concentration; LIP = Lipid concentration. Mean values ± standard deviation is reported.

| Sampling station | **MAN** | **PORT** | **DS** | **LR** | **API** |
| --- | --- | --- | --- | --- | --- |
| Phaeo (μg g^-1^) | 87.4 ± 22.0 | 19.8 ± 4.2 | 51.7 ± 2.1 | 202.6 ± 52.9 | 31.9 ± 6.2 |
| CPE (μg g^-1^) | 101.3 ± 27.2 | 22.0 ± 4.5 | 59.8 ± 1.8 | 219.6 ± 57.4 | 34.8 ± 5.9 |
| PRT (mg g^-1^) | 12.8 ± 3.3 | 9.8 ± 3.0 | 6.8 ± 0.3 | 5.2 ± 0.6 | 4.2 ± 1.0 |
| CHO (mg g^-1^) | 2.3 ± 0.3 | 0.3 ± 0.0 | 1.8 ± 0.7 | 1.0 ± 0.2 | 0.3 ± 0.1 |
| LIP (mg g^-1^) | 4.0 ± 0.3 | 1.0 ± 0.1 | 0.6 ± 0.1 | 1.0 ± 0.04 | 0.2 ± 0.0 |

**Table S4.** **Concentration (in μg g^-1^) of selected Heavy Metals Elements (HMs) and Polycyclic aromatic hydrocarbons (PAHs) detected in the sediment samples.** In bold values exceeding the lower threshold level (L1) specified in the Ministerial Decree 173/2016, in bold and italic those values exceeding also the upper threshold level (L2).

| Sampling station | | **MAN** | **PORT** | **DS** | **LR** | **API** |
| --- | --- | --- | --- | --- | --- | --- |
| **HMs** | Arsenic | 3.628 | 3.193 | 2.941 | 2.882 | 2.386 |
|  | Cadmium | 0.1915 | 0.0773 | 0.0533 | 0.0523 | 0.0493 |
|  | Chromium | 23.07 | 22.14 | 26.54 | 21.17 | 19.28 |
|  | Copper | **46.313** | 3.974 | 7.866 | 5.331 | 3.393 |
|  | Iron | 19119 | 12332 | 16709 | 15666 | 15937 |
|  | Mercury | 0.06835 | 0.02630 | 0.01865 | 0.01117 | 0.02761 |
|  | Manganese | 355.7 | 508.0 | 449.2 | 507.0 | 558.4 |
|  | Nickel | 3.911 | 2.786 | 3.555 | 3.539 | 3.703 |
|  | Lead | 19.912 | 14.835 | 10.569 | 11.799 | 8.348 |
|  | Vanadium | 28.31 | 18.99 | 24.90 | 24.83 | 26.09 |
|  | Zinc | ***297.15*** | **114.94** | 58.78 | 67.70 | 47.03 |
| **PAHs** | Naphthalene | **64.483** | 21.611 | **60.266** | **63.414** | 30.370 |
|  | 1-Methylnaphtalene | 51.412 | 21.632 | 29.975 | 36.011 | 17.875 |
|  | 2-Methylnaphtalene | 50.917 | 24.928 | 27.438 | 31.036 | 13.150 |
|  | Acenaphthylene | 8.267 | <0.5 | <0.5 | <0.5 | <0.5 |
|  | Acenaphthene | <0.5 | <0.5 | <0.5 | <0.5 | <0.5 |
|  | Fluorene | <0.5 | 1.578 | 1.280 | 1.881 | 1.428 |
|  | Phenanthrene | 12.184 | 7.579 | 9.029 | 10.800 | 5.302 |
|  | Anthracene | <0.05 | 0.166 | <0.05 | 0.123 | 0.163 |
|  | Fluoranthene | 17.404 | 6.305 | <0.05 | 0.861 | 4.022 |
|  | Pyrene | 4.039 | 0.787 | 0.782 | 0.556 | 0.172 |
|  | Benz[*a*]anthracene | <0.05 | <0.05 | <0.05 | <0.05 | <0.05 |
|  | Chrysene | <0.05 | <0.05 | <0.05 | <0.05 | <0.05 |
|  | Benzo[*b*]fluoranthene | 3.027 | 3.363 | 1.814 | 3.004 | 0.475 |
|  | Benzo[*k*]fluoranthene | 0.308 | 0.373 | 1.220 | 1.114 | 0.322 |
|  | Benzo[*a*]pyrene | 1.145 | 0.332 | 0.281 | 0.882 | 0.099 |
|  | 7,12-Dimethylbenz[*a*]anthracene | <0.5 | <0.5 | <0.5 | <0.5 | <0.5 |
|  | Benzo[*ghi*]perylene | 0.257 | 0.376 | 0.595 | 0.326 | <0.05 |
|  | Indeno[1,2,3-*cd*]pyrene | <0.05 | <0.05 | <0.05 | <0.05 | <0.05 |
|  | Dibenz[*a*,*h*]anthracene | <0.05 | <0.05 | 0.411 | <0.05 | <0.05 |
|  | Σ_8_ LMW PAHs | 187.26 | 77.49 | 127.99 | 143.27 | 68.29 |
|  | Σ_11_ HMW PAHs | 26.18 | 11.54 | 5.10 | 6.74 | 5.09 |
|  | Σ_19_ PAHs | 213.44 | 89.03 | 133.09 | 150.01 | 73.38 |

LMW = Low Molecular Weight compounds (Naphthalene, 1-Methylnaphtalene, 2-Methylnaphtalene, Acenaphthylene, Acenaphthene, Fluorene, Phenanthrene, Anthracene); HMW = High Molecular Weight compounds (Fluoranthene, Pyrene, Benz[*a*]anthracene, Chrysene, Benzo[*b*]fluoranthene, Benzo[*k*]fluoranthene, Benzo[*a*]pyrene, 7,12-Dimethylbenz[*a*]anthracene, Benzo[*ghi*]perylene, Indeno[1,2,3-*cd*]pyrene, Dibenz[*a*,*h*]anthracene).

**Table S5.** **Meio-and macrofauna univariate measures**. Total meiofauna and macrofauna abundance (N), number of taxa (S), Shannon’s diversity index (H’, based on log_2_), and Pielou’s equitability index (J’), characterizing the sediment at the investigated sampling stations. Mean values ± standard deviation are reported.

| Sampling station | **MAN** | **PORT** | **DS** | **LR** | **API** |
| --- | --- | --- | --- | --- | --- |
| **Meiofauna** | | | | | |
| Total abundance (no. of individuals 10 cm^-2^) | 816 ± 71 | 50 ± 8 | 2449 ± 105 | 1806 ± 58 | 451 ± 32 |
| Number of taxa | 9 ± 1 | 5 ± 1 | 10 ± 1 | 10 ± 2 | 8 ± 1 |
| Shannon’s diversity index | 1.35 ± 0.10 | 0.77 ± 0.15 | 0.36 ± 0.06 | 0.58 ± 0.05 | 0.72 ± 0.10 |
| Pielou’s equitability index | 0.43 ± 0.02 | 0.33 ± 0.06 | 0.11 ± 0.01 | 0.18 ± 0.01 | 0.24 ± 0.03 |
| **Macrofauna** | | | | | |
| Total abundance (no. of individuals m^-2^) | 607 ± 241 | 1214 ± 1051 | 880 ± 581 | 3449 ± 425 | 1491 ± 327 |
| Number of taxa | 12 ± 2 | 17 ± 3 | 19 ± 9 | 27 ± 5 | 24 ± 4 |
| Shannon’s diversity index | 2.85 ± 0.29 | 3.31 ± 0.33 | 3.19 ± 0.47 | 2.38 ± 0.56 | 3.55 ± 0.26 |
| Pielou’s equitability index | 0.79 ± 0.08 | 0.81 ± 0.11 | 0.78 ± 0.05 | 0.50 ± 0.09 | 0.78 ± 0.04 |

**Table S6.** **Similarity Percentages (SIMPER) test on meiofaunal community composition** **characterizing the sediment at the investigated sampling stations**. The contribution of taxa to the average dissimilarity is reported.

| **Groups MAN, PORT** | | | | | | |
| --- | --- | --- | --- | --- | --- | --- |
| Average dissimilarity = 49.26 | | | | | | |
| **Species** | **MAN** | **PORT** |  |  |  |  |
|  | Av. Abund. | Av. Abund. | Av. Diss. | Diss./SD | Contrib.% | Cum.% |
| Ciliata | 2.17 | 0.95 | 4.53 | 9.59 | 9.19 | 84.28 |
| Copepoda | 2.97 | 0.69 | 8.49 | 3.81 | 17.23 | 35.31 |
| Foraminifera | 2.82 | 1.16 | 6.13 | 7.45 | 12.45 | 75.09 |
| Nematoda | 4.97 | 2.56 | 8.91 | 11.48 | 18.08 | 18.08 |
| Oligochaeta | 1.71 | 0 | 6.30 | 21.97 | 12.79 | 62.64 |
| Ostracoda | 0.95 | 0 | 3.49 | 16.99 | 7.09 | 91.37 |
| Polychaeta | 2.22 | 0.30 | 7.16 | 3.83 | 14.54 | 49.85 |
| **Groups MAN, LR** | | | | | | |
| Average dissimilarity = 27.23 | | | | | | |
| **Species** | **MAN** | **LR** |  |  |  |  |
|  | Av. Abund. | Av. Abund. | Av. Diss. | Diss./SD | Contrib.% | Cum.% |
| Bivalvia | 0.30 | 1.10 | 1.95 | 1.75 | 7.18 | 83.40 |
| Ciliata | 2.17 | 0.81 | 3.29 | 2.12 | 12.10 | 39.72 |
| Foraminifera | 2.82 | 2.18 | 1.54 | 10.73 | 5.65 | 94.74 |
| Kinorhyncha | 0 | 1.26 | 3.02 | 7.84 | 11.09 | 50.80 |
| *Nauplii* | 1.47 | 2.36 | 2.14 | 5.79 | 7.86 | 76.22 |
| Nematoda | 4.97 | 6.39 | 3.42 | 9.52 | 12.55 | 27.62 |
| Oligochaeta | 1.71 | 0 | 4.10 | 20.35 | 15.07 | 15.07 |
| Ostracoda | 0.95 | 0 | 2.27 | 16.09 | 8.35 | 68.36 |
| Platyhelminthes | 0.65 | 1.24 | 1.55 | 1.16 | 5.69 | 89.09 |
| Sipuncula | 0 | 1.04 | 2.51 | 7.62 | 9.21 | 60.01 |
| **Groups MAN, DS** | | | | | | |
| Average dissimilarity = 25.78 | | | | | | |
| **Species** | **MAN** | **DS** |  |  |  |  |
|  | Av. Abund. | Av. Abund. | Av. Diss. | Diss./SD | Contrib.% | Cum.% |
| Ciliata | 2.17 | 1.13 | 2.58 | 7.36 | 10.01 | 55.61 |
| Copepoda | 2.97 | 2.22 | 1.84 | 6.18 | 7.14 | 62.75 |
| Kinorhyncha | 0 | 1.07 | 2.65 | 5.62 | 10.28 | 45.60 |
| *Nauplii* | 1.47 | 2.08 | 1.50 | 3.66 | 5.81 | 74.67 |
| Nematoda | 4.97 | 6.95 | 4.90 | 12.35 | 19.00 | 19.00 |
| Oligochaeta | 1.71 | 0 | 4.21 | 18.07 | 16.33 | 35.33 |
| Platyhelminthes | 0.65 | 0.30 | 1.35 | 1.12 | 5.25 | 85.50 |
| Polychaeta | 2.22 | 1.69 | 1.31 | 6.46 | 5.10 | 90.60 |
| Sipuncula | 0 | 0.65 | 1.58 | 1.31 | 6.11 | 68.86 |
| Tardigrada | 0 | 0.59 | 1.44 | 1.33 | 5.58 | 80.25 |
| **Groups MAN, API** | | | | | | |
| Average dissimilarity = 26.86 | | | | | | |
| **Species** | **MAN** | **API** |  |  |  |  |
|  | Av. Abund. | Av. Abund. | Av. Diss. | Diss./SD | Contrib.% | Cum.% |
| Bivalvia | 0.30 | 0.65 | 1.57 | 1.11 | 5.83 | 88.07 |
| Ciliata | 2.17 | 1.38 | 2.22 | 3.85 | 8.26 | 76.27 |
| Copepoda | 2.97 | 1.79 | 3.34 | 6.60 | 12.43 | 47.73 |
| Foraminifera | 2.82 | 1.83 | 2.79 | 6.64 | 10.37 | 58.10 |
| Kinorhyncha | 0 | 1.66 | 4.68 | 16.01 | 17.43 | 35.30 |
| Nematoda | 4.97 | 4.49 | 1.35 | 4.40 | 5.04 | 93.11 |
| Oligochaeta | 1.71 | 0 | 4.80 | 21.92 | 17.87 | 17.87 |
| Ostracoda | 0.95 | 0 | 2.66 | 16.89 | 9.90 | 68.00 |
| Polychaeta | 2.22 | 1.65 | 1.60 | 5.45 | 5.97 | 82.24 |
| **Groups PORT, LR** | | | | | | |
| Average dissimilarity = 53.71 | | | | | | |
| **Species** | **PORT** | **LR** |  |  |  |  |
|  | Av. Abund. | Av. Abund. | Av. Diss. | Diss./SD | Contrib.% | Cum.% |
| Bivalvia | 0 | 1.10 | 3.89 | 20.56 | 7.24 | 83.06 |
| Copepoda | 0.69 | 2.57 | 6.72 | 3.22 | 12.52 | 51.87 |
| Foraminifera | 1.16 | 2.18 | 3.61 | 4.92 | 6.72 | 96.67 |
| Kinorhyncha | 0 | 1.26 | 4.44 | 8.19 | 8.27 | 60.14 |
| *Nauplii* | 1.23 | 2.36 | 4.02 | 9.32 | 7.48 | 75.83 |
| Nematoda | 2.56 | 6.39 | 13.57 | 13.92 | 25.27 | 25.27 |
| Platyhelminthes | 0 | 1.24 | 4.41 | 4.28 | 8.21 | 68.35 |
| Polychaeta | 0.30 | 2.42 | 7.56 | 4.19 | 14.08 | 39.35 |
| Sipuncula | 0 | 1.04 | 3.70 | 7.39 | 6.88 | 89.94 |
| **Groups PORT, DS** | | | | | | |
| Average dissimilarity = 49.49 | | | | | | |
| **Species** | **PORT** | **DS** |  |  |  |  |
|  | Av. Abund. | Av. Abund. | Av. Diss. | Diss./SD | Contrib.% | Cum.% |
| Copepoda | 0.69 | 2.22 | 5.71 | 2.62 | 11.54 | 44.23 |
| Foraminifera | 1.16 | 2.72 | 5.70 | 6.01 | 11.51 | 55.74 |
| Kinorhyncha | 0 | 1.07 | 3.96 | 5.23 | 8.00 | 74.19 |
| *Nauplii* | 1.23 | 2.08 | 3.13 | 6.29 | 6.33 | 80.52 |
| Nematoda | 2.56 | 6.95 | 16.18 | 13.91 | 32.69 | 32.69 |
| Ostracoda | 0 | 0.65 | 2.47 | 1.30 | 4.99 | 85.51 |
| Polychaeta | 0.30 | 1.69 | 5.17 | 2.83 | 10.45 | 66.19 |
| Sipuncula | 0 | 0.65 | 2.33 | 1.31 | 4.71 | 90.22 |
| **Groups PORT, API** | | | | | | |
| Average dissimilarity = 38.44 | | | | | | |
| **Species** | **PORT** | **API** |  |  |  |  |
|  | Av. Abund. | Av. Abund. | Av. Diss. | Diss./SD | Contrib.% | Cum.% |
| Bivalvia | 0 | 0.65 | 2.92 | 1.30 | 7.59 | 86.63 |
| Copepoda | 0.69 | 1.79 | 5.01 | 1.88 | 13.03 | 71.21 |
| Foraminifera | 1.16 | 1.83 | 3.01 | 2.80 | 7.83 | 79.04 |
| Kinorhyncha | 0 | 1.66 | 7.49 | 15.79 | 19.50 | 42.12 |
| Nematoda | 2.56 | 4.49 | 8.69 | 9.62 | 22.62 | 22.62 |
| Platyhelminthes | 0 | 0.59 | 2.64 | 1.33 | 6.86 | 93.49 |
| Polychaeta | 0.30 | 1.65 | 6.17 | 2.78 | 16.06 | 58.17 |
| **Groups LR, DS** | | | | | | |
| Average dissimilarity = 15.84 | | | | | | |
| **Species** | **LR** | **DS** |  |  |  |  |
|  | Av. Abund. | Av. Abund. | Av. Diss. | Diss./SD | Contrib.% | Cum.% |
| Bivalvia | 1.10 | 0.30 | 1.95 | 1.75 | 12.28 | 26.80 |
| Ciliata | 0.81 | 1.13 | 1.11 | 0.89 | 7.03 | 80.33 |
| Copepoda | 2.57 | 2.22 | 0.83 | 9.18 | 5.26 | 92.26 |
| Foraminifera | 2.18 | 2.72 | 1.27 | 2.49 | 8.03 | 73.30 |
| Nematoda | 6.39 | 6.95 | 1.35 | 7.86 | 8.54 | 65.27 |
| Ostracoda | 0 | 0.65 | 1.59 | 1.31 | 10.05 | 47.91 |
| Platyhelminthes | 1.24 | 0.30 | 2.30 | 1.75 | 14.51 | 14.51 |
| Polychaeta | 2.42 | 1.69 | 1.75 | 8.01 | 11.06 | 37.86 |
| Sipuncula | 1.04 | 0.65 | 1.06 | 0.89 | 6.67 | 87.00 |
| Tardigrada | 0 | 0.59 | 1.40 | 1.33 | 8.82 | 56.73 |
| **Groups LR, API** | | | | | | |
| Average dissimilarity = 21.87 | | | | | | |
| **Species** | **LR** | **API** |  |  |  |  |
|  | Av. Abund. | Av. Abund. | Av. Diss. | Diss./SD | Contrib.% | Cum.% |
| Bivalvia | 1.10 | 0.65 | 1.22 | 0.89 | 5.59 | 90.55 |
| Ciliata | 0.81 | 1.38 | 1.64 | 0.95 | 7.49 | 84.97 |
| Copepoda | 2.57 | 1.79 | 2.15 | 5.39 | 9.82 | 59.75 |
| *Nauplii* | 2.36 | 1.29 | 2.90 | 7.87 | 13.25 | 36.94 |
| Nematoda | 6.39 | 4.49 | 5.18 | 14.87 | 23.69 | 23.69 |
| Platyhelminthes | 1.24 | 0.59 | 1.79 | 1.20 | 8.17 | 77.47 |
| Polychaeta | 2.42 | 1.65 | 2.09 | 6.68 | 9.56 | 69.31 |
| Sipuncula | 1.04 | 0 | 2.84 | 7.72 | 12.99 | 49.92 |
| **Groups DS, API** | | | | | | |
| Average dissimilarity = 23.75 | | | | | | |
| **Species** | **DS** | **API** |  |  |  |  |
|  | Av. Abund. | Av. Abund. | Av. Diss. | Diss./SD | Contrib.% | Cum.% |
| Bivalvia | 0.30 | 0.65 | 1.56 | 1.11 | 6.56 | 84.51 |
| Foraminifera | 2.72 | 1.83 | 2.47 | 3.68 | 10.39 | 39.51 |
| Kinorhyncha | 1.07 | 1.66 | 1.65 | 3.37 | 6.95 | 71.08 |
| *Nauplii* | 2.08 | 1.29 | 2.19 | 5.04 | 9.22 | 48.74 |
| Nematoda | 6.95 | 4.49 | 6.92 | 18.94 | 29.12 | 29.12 |
| Ostracoda | 0.65 | 0 | 1.87 | 1.31 | 7.87 | 56.60 |
| Platyhelminthes | 0.30 | 0.59 | 1.40 | 1.05 | 5.87 | 90.38 |
| Sipuncula | 0.65 | 0 | 1.79 | 1.31 | 7.52 | 64.13 |
| Tardigrada | 0.59 | 0 | 1.63 | 1.33 | 6.86 | 77.94 |

Av. Abund. = Average Abundance; Av. Diss. = Average Dissimilarity; Diss. = Dissimilarity; SD = Standard Deviation; Contrib. = Contribution; Cum. = Cumulative contribution.

**Table S7.** **Spearman’s rank correlation between meiofauna (-me) and macrofauna (-ma) descriptors** **and environmental features**. S = taxa richness; N = total abundance; J’ = Pielou’s equitability index; H’ = Shannon’s diversity index, based on log_2_ and (a) organic matter quantity and quality ~~and microbial abundance~~; (b) Heavy Metals (HMs), Σ_19_ Polycyclic aromatic hydrocarbons (PAHs), and grain size (sand% and silt/mud%). In italics significant correlations with *p* <0.05; in bold significant correlations with *p* <0.01.

| **a)** | **S-me** | **N-me** | **J’-me** | **H’-me** | **S-ma** | **N-ma** | **J’-ma** | **H’-ma** | **TPN** | **Chl-*a*** | **Phaeo** | **CPE** | **PRT** | **CHO** | **PRT/CHO** | **LIP** | **BPC** |
| --- | --- | --- | --- | --- | --- | --- | --- | --- | --- | --- | --- | --- | --- | --- | --- | --- | --- |
| **S-me** | 1 |  |  |  |  |  |  |  |  |  |  |  |  |  |  |  |  |
| **N-me** | 0.7245 | 1 |  |  |  |  |  |  |  |  |  |  |  |  |  |  |  |
| **J’-me** | -0.3974 | -0.7244 | 1 |  |  |  |  |  |  |  |  |  |  |  |  |  |  |
| **H’-me** | -0.0627 | -0.5412 | 0.9320 | 1 |  |  |  |  |  |  |  |  |  |  |  |  |  |
| **S-ma** | 0.3676 | 0.2545 | -0.5400 | -0.4759 | 1 |  |  |  |  |  |  |  |  |  |  |  |  |
| **N-ma** | 0.3188 | 0.2252 | -0.3584 | -0.2826 | 0.7272 | 1 |  |  |  |  |  |  |  |  |  |  |  |
| **J’-ma** | -0.4265 | -0.3910 | 0.3322 | 0.2138 | -0.5433 | -0.9188 | 1 |  |  |  |  |  |  |  |  |  |  |
| **H’-ma** | -0.2497 | -0.3371 | 0.0155 | -0.0865 | 0.1515 | -0.4971 | 0.7319 | 1 |  |  |  |  |  |  |  |  |  |
| **~~TPN~~** | ~~-0.0936~~ | ~~-0.4504~~ | *~~0.6890~~* | **~~0.7160~~** | ~~-0.5272~~ | *~~-0.6208~~* | *~~0.6468~~* | ~~0.3514~~ | ~~1~~ |  |  |  |  |  |  |  |  |
| **Chl-*a*** | *0.6232* | **0.7116** | -0.1760 | 0.0259 | -0.1677 | -0.2591 | 0.0252 | -0.1441 | ~~0.0273~~ | 1 |  |  |  |  |  |  |  |
| **Phaeo** | *0.5413* | **0.7840** | -0.4510 | -0.3157 | -0.0669 | -0.2886 | 0.1038 | 0.0399 | ~~-0.1289~~ | 0.8884 | 1 |  |  |  |  |  |  |
| **CPE** | *0.5530* | **0.7847** | -0.4315 | -0.2893 | -0.0761 | -0.2887 | 0.0980 | 0.0246 | ~~-0.1167~~ | 0.9057 | 0.9992 | 1 |  |  |  |  |  |
| **PRT** | -0.2456 | -0.3321 | **0.7614** | **0.7229** | -0.5393 | -0.1981 | 0.1011 | -0.3145 | ~~0.4269~~ | 0.0542 | -0.1849 | -0.1662 | 1 |  |  |  |  |
| **CHO** | *0.5583* | 0.3876 | 0.2267 | 0.4535 | -0.1222 | 0.0808 | -0.3255 | -0.5395 | ~~0.1348~~ | 0.6069 | 0.2470 | 0.2798 | 0.4425 | 1 |  |  |  |
| **PRT/CHO** | **-0.7974** | *-0.6536* | 0.3556 | 0.0694 | -0.1256 | -0.0548 | 0.2408 | 0.2502 | ~~0.0891~~ | -0.5776 | -0.4633 | -0.4771 | 0.3059 | -0.6113 | 1 |  |  |
| **LIP** | 0.0960 | -0.1177 | **0.7172** | **0.8187** | *-0.6414* | -0.4476 | 0.2391 | -0.2288 | ~~0.6030~~ | 0.4379 | 0.1347 | 0.1618 | 0.7964 | 0.6754 | -0.1760 | 1 |  |
| **BPC** | -0.0494 | -0.1998 | **0.7538** | **0.7938** | *-0.5768* | -0.2721 | 0.1080 | -0.3417 | ~~0.4951~~ | 0.2636 | -0.0361 | -0.0109 | 0.9557 | 0.6362 | 0.0517 | 0.9309 | 1 |

continue…

…continue

| **b)** | **S-me** | **N-me** | **J’-me** | **H’-me** | **S-ma** | **N-ma** | **J’-ma** | **H’-ma** | **As** | **Cd** | **Cr** | **Cu** | **Fe** | **Hg** | **Mn** | **Ni** | **Pb** | **V** | **Zn** | **Σ_19_** **PAHs** | **sand%** | **silt/mud%** |
| --- | --- | --- | --- | --- | --- | --- | --- | --- | --- | --- | --- | --- | --- | --- | --- | --- | --- | --- | --- | --- | --- | --- |
| **S-me** | 1 |  |  |  |  |  |  |  |  |  |  |  |  |  |  |  |  |  |  |  |  |  |
| **N-me** | 0.7245 | 1 |  |  |  |  |  |  |  |  |  |  |  |  |  |  |  |  |  |  |  |  |
| **J’-me** | -0.3974 | -0.7244 | 1 |  |  |  |  |  |  |  |  |  |  |  |  |  |  |  |  |  |  |  |
| **H’-me** | -0.0627 | -0.5412 | 0.9320 | 1 |  |  |  |  |  |  |  |  |  |  |  |  |  |  |  |  |  |  |
| **S-ma** | 0.3676 | 0.2545 | -0.5400 | -0.4759 | 1 |  |  |  |  |  |  |  |  |  |  |  |  |  |  |  |  |  |
| **N-ma** | 0.3188 | 0.2252 | -0.3584 | -0.2826 | 0.7272 | 1 |  |  |  |  |  |  |  |  |  |  |  |  |  |  |  |  |
| **J’-ma** | -0.4265 | -0.3910 | 0.3322 | 0.2138 | -0.5433 | -0.9188 | 1 |  |  |  |  |  |  |  |  |  |  |  |  |  |  |  |
| **H’-ma** | -0.2497 | -0.3371 | 0.0155 | -0.0865 | 0.1515 | -0.4971 | 0.7319 | 1 |  |  |  |  |  |  |  |  |  |  |  |  |  |  |
| **As** | 0.0672 | -0.0472 | 0.3109 | 0.3628 | -0.2442 | -0.1108 | 0.1208 | -0.0503 | 1 |  |  |  |  |  |  |  |  |  |  |  |  |  |
| **Cd** | 0.0661 | -0.2520 | **0.7437** | **0.8470** | *-0.5907* | -0.4189 | 0.2956 | -0.1209 | 0.7123 | 1 |  |  |  |  |  |  |  |  |  |  |  |  |
| **Cr** | 0.2723 | 0.1880 | -0.1098 | -0.0209 | 0.2394 | 0.4283 | -0.3481 | -0.2377 | 0.7975 | 0.2807 | 1 |  |  |  |  |  |  |  |  |  |  |  |
| **Cu** | 0.2439 | -0.0982 | *0.6717* | **0.8333** | *-0.5442* | -0.3496 | 0.1751 | -0.2416 | 0.5970 | 0.9680 | 0.2262 | 1 |  |  |  |  |  |  |  |  |  |  |
| **Fe** | *0.5804* | 0.2193 | 0.0219 | 0.2624 | 0.0560 | 0.0621 | -0.0720 | -0.0672 | 0.7050 | 0.5578 | 0.7252 | 0.5844 | 1 |  |  |  |  |  |  |  |  |  |
| **Hg** | 0.0244 | -0.4166 | **0.7935** | **0.9007** | -0.5205 | -0.3772 | 0.3034 | -0.0479 | 0.6120 | 0.9614 | 0.2230 | 0.9310 | 0.5645 | 1 |  |  |  |  |  |  |  |  |
| **Mn** | -0.0631 | -0.0491 | -0.4791 | -0.5258 | 0.4467 | 0.1276 | 0.1315 | 0.5365 | 0.1954 | -0.3778 | 0.3970 | -0.5061 | 0.2632 | -0.3149 | 1 |  |  |  |  |  |  |  |
| **Ni** | *0.5570* | 0.2128 | -0.1106 | 0.1141 | 0.1557 | 0.0571 | -0.0170 | 0.0835 | 0.6292 | 0.4064 | 0.6907 | 0.4170 | 0.9732 | 0.4362 | 0.4566 | 1 |  |  |  |  |  |  |
| **Pb** | -0.0526 | -0.1945 | *0.6021* | *0.6353* | -0.5375 | -0.3823 | 0.3123 | -0.0514 | 0.9049 | 0.9067 | 0.4914 | 0.8020 | 0.5369 | 0.8018 | -0.1365 | 0.4126 | 1 |  |  |  |  |  |
| **V** | *0.5778* | 0.2126 | -0.0680 | 0.1689 | 0.1158 | 0.0273 | -0.0069 | 0.0618 | 0.6231 | 0.4542 | 0.6598 | 0.4756 | 0.9820 | 0.4843 | 0.3918 | 0.9969 | 0.4336 | 1 |  |  |  |  |
| **Zn** | 0.0226 | -0.2540 | **0.7585** | **0.8429** | *-0.6293* | -0.4517 | 0.3188 | -0.1227 | 0.7026 | 0.9961 | 0.2437 | 0.9575 | 0.4953 | 0.9418 | -0.4131 | 0.3388 | 0.9169 | 0.3869 | 1 |  |  |  |
| **Σ_19_ PAHs** | 0.5099 | 0.3635 | 0.2401 | 0.4427 | -0.3428 | -0.1930 | 0.0079 | -0.3005 | 0.7523 | 0.7910 | 0.5265 | 0.8350 | 0.7526 | 0.6521 | -0.2637 | 0.6161 | 0.7854 | 0.6518 | 0.7801 | 1 |  |  |
| **sand%** | 0.4843 | *0.6213* | **-0.9176** | **-0.8122** | *0.6635* | 0.4930 | -0.4394 | -0.0367 | -0.5155 | -0.7938 | -0.0088 | -0.6697 | -0.0270 | -0.7609 | 0.3929 | 0.1055 | -0.7866 | 0.0737 | -0.8275 | -0.3600 | 1 |  |
| **silt/mud%** | -0.4843 | *-0.6213* | **0.9176** | **0.8122** | *-0.6635* | -0.4930 | 0.4394 | 0.0367 | 0.5155 | 0.7938 | 0.0088 | 0.6697 | 0.0270 | 0.7609 | -0.3929 | -0.1055 | 0.7866 | -0.0737 | 0.8275 | 0.3600 | -1 | 1 |

~~TPN = Total Prokaryotic Number;~~ Chl-*a* = Chlorophyll-*a* concentration; Phaeo = Phaeopigment concentration; CPE = chloroplast pigment equivalents; PRT = Protein concentration; CHO = Carbohydrate concentration; PRT/CHO = Protein to carbohydrate ratio; LIP = Lipid concentration; BPC = Biopolymeric organic carbon.

**Table S8.** **List of macrofauna species and their relative abundances (no. of individuals m^2^), characterizing the sediment at the investigated sampling stations.**

| **Taxon** | **MAN** | **PORT** | **DS** | **LR** | **API** |
| --- | --- | --- | --- | --- | --- |
| **Anellida** | | | | | |
| *Ampharete* sp |  |  | 3.5 |  |  |
| *Aponuphis bilineata* |  |  |  |  | 24.6 |
| *Aricidea (Aricidea) fragilis* |  | 35.1 | 42.1 | 14.0 | 80.7 |
| *Capitella capitata* | 38.6 | 101.8 | 49.1 | 249.1 | 98.2 |
| *Chaetozone caputesocis* | 49.1 |  | 14.0 |  |  |
| Cirratulidae nd | 10.5 |  |  |  |  |
| *Cossura soyeri* |  |  |  | 35.1 |  |
| *Diplocirrus glaucus* |  |  | 3.5 |  |  |
| *Euclymene oerstedii* |  |  |  |  | 49.1 |
| *Exogone verugera* |  |  |  |  | 7.0 |
| *Gallardoneris iberica* |  |  | 21.1 | 3.5 | 10.5 |
| *Glycera capitata* |  |  | 7.0 | 3.5 | 7.0 |
| *Glycera fallax* |  |  |  |  | 7.0 |
| *Glycera unicornis* |  | 7.0 |  |  |  |
| *Harmothoe* sp |  |  |  | 7.0 |  |
| *Heteromastus filiformis* | 10.5 | 164.9 | 3.5 | 59.6 | 14.0 |
| *Hilbigneris gracilis* |  |  |  | 3.5 | 3.5 |
| *Jasmineira caudata* |  |  |  |  | 150.9 |
| *Jasmineira elegans* | 157.9 | 122.8 |  |  | 38.6 |
| *Lagis koreni* |  |  | 7.0 | 10.5 |  |
| *Lumbrineris latreilli* |  | 24.6 |  | 7.0 | 49.1 |
| *Magelona filiformis* |  |  |  |  | 10.5 |
| *Melinna palmata* |  |  | 7.0 | 3.5 | 31.6 |
| *Micronephthys longicornis* |  | 10.5 | 10.5 | 7.0 | 42.1 |
| *Nephtys hombergii* | 7.0 |  |  |  |  |
| *Nephtys incisa* |  |  |  | 10.5 |  |
| *Nereis* sp |  |  | 3.5 |  | 7.0 |
| *Ophelina* sp |  | 3.5 |  | 7.0 |  |
| *Paraonis fulgens* |  | 38.6 |  | 7.0 |  |
| *Paradoneis armata* |  | 21.1 | 66.7 | 17.5 | 24.6 |
| Phyllodocidae nd |  | 7.0 |  |  | 10.5 |
| *Poecilochaetus serpens* |  |  | 3.5 |  |  |
| *Polydora paucibranchis* | 10.5 | 10.5 |  |  |  |
| *Prionospio cirrifera* |  | 21.1 |  |  | 115.8 |
| *Prionospio* sp |  |  |  | 108.8 | 115.8 |
| *Sabellaria spinulosa* |  |  |  | 14.0 |  |
| Spionidae nd |  |  | 7.0 |  |  |
| *Spiophanes bombyx* | 3.5 |  |  |  | 301.8 |
| *Spirobranchus triqueter* | 3.5 |  |  | 17.5 |  |
| *Sternaspis scutata* |  | 14.0 | 287.7 | 45.6 |  |
| *Streblospio* sp | 115.8 |  |  |  |  |
| *Tharyx* sp | 52.6 |  | 10.5 | 17.5 | 7.0 |
| *Tubificoides swirencoides* | 87.7 | 407.0 | 101.8 | 1701.8 |  |
| **Anfipoda** | | | | | |
| *Ampelisca diadema* |  |  | 10.5 |  | 45.6 |
| *Ampelisca spinipes* |  |  | 7.0 | 7.0 |  |
| *Aora gracilis* | 3.5 |  |  | 7.0 |  |
| *Leptocheirus pilosus* |  |  | 38.6 |  |  |
| *Pariambus typicus* |  | 7.0 |  |  |  |
| *Phtisica marina* |  | 35.1 | 28.1 | 42.1 | 3.5 |
| **Anthozoa** | | | | | |
| Actiniaria nd | 3.5 |  |  |  |  |
| **Bivalvia** | | | | | |
| *Abra alba* |  |  | 49.1 | 73.7 | 7.0 |
| *Abra prismatica* | 3.5 |  |  |  |  |
| *Abra tenuis* | 3.5 |  |  |  |  |
| *Anadara transversa* |  |  | 3.5 | 112.3 | 3.5 |
| *Arcuatula senhousia* | 3.5 | 3.5 |  | 7.0 |  |
| *Bosemprella incarnata* |  |  |  |  | 14.0 |
| *Ctena decussata* | 7.0 |  |  |  |  |
| *Hiatella arctica* |  |  |  | 17.5 |  |
| *Kurtiella bidentata* |  | 3.5 |  | 21.1 | 143.9 |
| *Moerella distorta* |  |  | 3.5 |  |  |
| *Musculus subpictus* |  |  |  | 10.5 |  |
| *Mytilus galloprovincialis* |  |  | 7.0 | 568.4 |  |
| *Nucula nitidosa* |  | 38.6 | 7.0 | 14.0 | 7.0 |
| *Peronidia albicans* |  | 3.5 | 7.0 |  | 7.0 |
| *Pitar rudis* |  |  | 10.5 | 3.5 | 10.5 |
| *Pododesmus patelliformis* |  |  |  | 3.5 |  |
| *Polititapes aureus* |  | 3.5 |  |  |  |
| *Spisula subtruncata* |  |  | 3.5 |  |  |
| *Thracia* sp |  | 3.5 |  |  |  |
| *Thyasira* sp |  |  |  | 3.5 |  |
| *Varicorbula gibba* |  | 7.0 |  | 3.5 |  |
| *Venus* sp |  | 7.0 |  |  | 3.5 |
| **Bryozoa** | | | | | |
| *Amathia semiconvoluta* | 7.0 |  | 3.5 |  |  |
| *Bugula neritina* |  | 3.5 |  | 3.5 |  |
| **Cumacea** | | | | | |
| *Iphinoe adriatica* |  | 17.5 | 3.5 |  | 3.5 |
| *Pseudocuma (Pseudocuma) longicorne* |  | 14.0 |  |  |  |
| **Gastropoda** | | | | | |
| *Aporrhais pespelicani* |  |  | 3.5 |  | 3.5 |
| *Bittium reticulatum* |  | 3.5 |  | 10.5 |  |
| *Chrysallida* sp |  | 7.0 |  | 42.1 |  |
| *Hyala vitrea* |  | 7.0 |  | 14.0 |  |
| *Ocenebra edwardsii* |  | 3.5 |  |  |  |
| *Tritia nitida* | 3.5 |  | 3.5 | 52.6 | 3.5 |
| **Hydrozoa** | | | | | |
| Hydrozoa nd | 3.5 |  |  |  |  |
| **Isopoda** | | | | | |
| Janiridae nd |  |  |  | 7.0 |  |
| **Nematoda** | | | | | |
| *Anticoma* sp | 3.5 |  |  |  |  |
| *Elzalia* sp | 3.5 |  |  | 3.5 |  |
| Nematoda nd | 7.0 |  |  |  |  |
| *Paracyatholaimus* sp | 3.5 |  |  |  |  |
| *Pareurystomina* sp | 3.5 |  |  | 7.0 |  |
| **Nemertea** | | | | | |
| Tubulanidae nd |  | 52.6 | 38.6 | 21.1 | 24.6 |
| **Ophiuroidea** | | | | | |
| *Ophiura albida* |  | 3.5 | 3.5 |  |  |
| **Scaphopoda** | | | | | |
| *Antalis inaequicostata* |  |  |  |  | 3.5 |
| **Tanaidacea** | | | | | |
| *Apseudopsis latreillii* |  |  |  | 42.1 |  |
| **Abundance** | **607** | **1214** | **880** | **3449** | **1491** |

**Table S9.** **Similarity Percentages (SIMPER) test on macrofaunal community composition characterizing the** **sampling stations**. The contribution of taxa to the average dissimilarity is reported.

| **Groups MAN, PORT** | | | | | | |
| --- | --- | --- | --- | --- | --- | --- |
| Average dissimilarity = 74.41 | | | | | | |
| **Species** | **MAN** | **PORT** |  |  |  |  |
|  | Av. Abund. | Av. Abund. | Av. Diss. | Diss./SD | Contrib.% | Cum.% |
| *Aricidea fragilis* | 0 | 1.80 | 2.35 | 1.31 | 3.16 | 30.92 |
| *Chaetozone caputesocis* | 2.52 | 0 | 3.58 | 3.55 | 4.81 | 15.81 |
| *Heteromastus filiformis* | 0.79 | 3.45 | 3.69 | 2.02 | 4.96 | 10.99 |
| *Lumbrineris latreilli* | 0 | 2.17 | 3.08 | 3.90 | 4.14 | 24.33 |
| Nematoda nd | 1.51 | 0 | 2.05 | 1.24 | 2.75 | 39.49 |
| Nemertea nd | 0 | 1.97 | 2.55 | 1.33 | 3.43 | 27.76 |
| *Paradoneis armata* | 0 | 1.59 | 2.22 | 1.28 | 2.99 | 33.91 |
| *Phtisica marina* | 0 | 2.36 | 3.26 | 9.26 | 4.38 | 20.19 |
| *Pseudocuma longicorne* | 0 | 1.40 | 2.11 | 1.32 | 2.83 | 36.74 |
| *Streblospio* sp | 3.20 | 0 | 4.49 | 6.33 | 6.03 | 6.03 |
| **Groups MAN, LR** | | | | | | |
| Average dissimilarity = 83.62 | | | | | | |
| **Species** | **MAN** | **LR** |  |  |  |  |
|  | Av. Abund. | Av. Abund. | Av. Diss. | Diss./SD | Contrib.% | Cum.% |
| *Abra alba* | 0 | 2.91 | 2.99 | 20.80 | 3.57 | 19.71 |
| *Apseudopsis latreillii* | 0 | 2.36 | 2.46 | 3.31 | 2.94 | 34.97 |
| *Chaetozone caputesocis* | 2.52 | 0 | 2.62 | 3.94 | 3.14 | 22.85 |
| *Chrysallida* sp | 0 | 2.51 | 2.62 | 5.04 | 3.14 | 25.99 |
| *Jasmineira elegans* | 3.26 | 0 | 3.35 | 3.98 | 4.01 | 8.30 |
| *Mytilus galloprovincialis* | 0 | 3.63 | 3.58 | 2.09 | 4.29 | 4.29 |
| *Phtisica marina* | 0 | 2.43 | 2.49 | 6.37 | 2.97 | 32.03 |
| *Prionospio* sp | 0 | 2.34 | 2.57 | 1.31 | 3.07 | 29.06 |
| *Streblospio* sp | 3.20 | 0 | 3.30 | 7.30 | 3.94 | 12.24 |
| *Tubificoides swirencoides* | 2.98 | 5.99 | 3.26 | 1.71 | 3.90 | 16.14 |
| **Groups MAN, DS** | | | | | | |
| Average dissimilarity = 84.73 | | | | | | |
| **Species** | **MAN** | **DS** |  |  |  |  |
|  | Av. Abund. | Av. Abund. | Av. Diss. | Diss./SD | Contrib.% | Cum.% |
| *Ampelisca diadema* | 0 | 1.82 | 2.69 | 3.16 | 3.18 | 36.93 |
| *Aricidea fragilis* | 0 | 2.28 | 3.25 | 3.60 | 3.83 | 33.76 |
| *Jasmineira elegans* | 3.26 | 0 | 4.77 | 2.63 | 5.64 | 12.22 |
| *Leptocheirus pilosus* | 0 | 2.46 | 3.54 | 4.85 | 4.18 | 21.94 |
| Nemertea nd | 0 | 2.41 | 3.38 | 8.74 | 3.99 | 29.92 |
| *Phtisica marina* | 0 | 2.15 | 3.38 | 1.79 | 3.99 | 25.94 |
| *Sternaspis scutata* | 0 | 3.97 | 5.58 | 8.49 | 6.59 | 6.59 |
| *Streblospio* sp | 3.20 | 0 | 4.70 | 3.16 | 5.55 | 17.77 |
| *Tharyx* sp | 1.18 | 1.32 | 2.35 | 0.98 | 2.77 | 42.77 |
| *Tubificoides swirencoides* | 2.98 | 2.25 | 2.60 | 0.97 | 3.07 | 40.00 |
| **Groups MAN, API** | | | | | | |
| Average dissimilarity = 88.30 | | | | | | |
| **Species** | **MAN** | **API** |  |  |  |  |
|  | Av. Abund. | Av. Abund. | Av. Diss. | Diss./SD | Contrib.% | Cum.% |
| *Ampelisca diadema* | 0 | 2.54 | 2.96 | 4.32 | 3.35 | 26.88 |
| *Aricidea fragilis* | 0 | 2.73 | 3.06 | 4.77 | 3.47 | 20.08 |
| *Chaetozone caputesocis* | 2.52 | 0 | 2.92 | 3.88 | 3.31 | 30.19 |
| *Euclymene oerstedii* | 0 | 2.63 | 3.04 | 6.45 | 3.45 | 23.52 |
| *Kurtiella bidentata* | 0 | 3.43 | 3.93 | 10.70 | 4.45 | 4.45 |
| *Lumbrineris latreilli* | 0 | 2.52 | 2.86 | 6.79 | 3.24 | 36.72 |
| *Micronephthys longicornis* | 0 | 2.54 | 2.91 | 16.7 | 3.30 | 33.49 |
| *Spiophanes bombyx* | 0.61 | 3.81 | 3.65 | 2.28 | 4.14 | 12.75 |
| *Streblospio* sp | 3.20 | 0 | 3.67 | 7.38 | 4.16 | 8.61 |
| *Tubificoides swirencoides* | 2.98 | 0 | 3.41 | 6.89 | 3.86 | 16.61 |
| **Groups PORT, LR** | | | | | | |
| Average dissimilarity = 63.81 | | | | | | |
| **Species** | **PORT** | **LR** |  |  |  |  |
|  | Av. Abund. | Av. Abund. | Av. Diss. | Diss./SD | Contrib.% | Cum.% |
| *Abra alba* | 0 | 2.91 | 2.62 | 11.45 | 4.11 | 13.25 |
| *Apseudopsis latreillii* | 0 | 2.36 | 2.15 | 3.26 | 3.38 | 27.45 |
| *Chrysallida* sp | 0.71 | 2.51 | 1.72 | 1.53 | 2.69 | 30.14 |
| *Cossura soyeri* | 0 | 1.79 | 1.71 | 1.31 | 2.68 | 32.82 |
| *Heteromastus filiformis* | 3.45 | 1.81 | 1.58 | 1.49 | 2.48 | 35.30 |
| *Jasmineira elegans* | 3.02 | 0 | 2.67 | 3.87 | 4.19 | 9.14 |
| *Mytilus galloprovincialis* | 0 | 3.63 | 3.16 | 2.02 | 4.95 | 4.95 |
| *Prionospio* sp | 0 | 2.34 | 2.24 | 1.30 | 3.51 | 24.07 |
| *Tritia nitida* | 0 | 2.52 | 2.26 | 5.13 | 3.54 | 20.56 |
| *Tubificoides swirencoides* | 3.86 | 5.99 | 2.41 | 1.42 | 3.77 | 17.03 |
| **Groups PORT, DS** | | | | | | |
| Average dissimilarity = 72.31 | | | | | | |
| **Species** | **PORT** | **DS** |  |  |  |  |
|  | Av. Abund. | Av. Abund. | Av. Diss. | Diss./SD | Contrib.% | Cum.% |
| *Abra alba* | 0 | 1.92 | 1.94 | 1.30 | 2.68 | 37.09 |
| *Ampelisca diadema* | 0 | 1.82 | 2.23 | 3.48 | 3.08 | 28.74 |
| *Capitella capitata* | 2.94 | 1.95 | 1.97 | 0.96 | 2.73 | 34.41 |
| *Heteromastus filiformis* | 3.45 | 0.61 | 3.54 | 2.02 | 4.89 | 9.83 |
| *Jasmineira elegans* | 3.02 | 0 | 3.57 | 3.01 | 4.94 | 4.94 |
| *Leptocheirus pilosus* | 0 | 2.46 | 2.94 | 5.27 | 4.07 | 18.08 |
| *Lumbrineris latreilli* | 2.17 | 0 | 2.67 | 2.77 | 3.70 | 25.66 |
| *Paradoneis armata* | 1.59 | 1.25 | 2.13 | 1.34 | 2.94 | 31.68 |
| *Sternaspis scutata* | 1.43 | 3.97 | 3.03 | 1.76 | 4.19 | 14.02 |
| *Tubificoides swirencoides* | 3.86 | 2.25 | 2.81 | 1.07 | 3.88 | 21.96 |
| **Groups PORT, API** | | | | | | |
| Average dissimilarity = 71.27 | | | | | | |
| **Species** | **PORT** | **API** |  |  |  |  |
|  | Av. Abund. | Av. Abund. | Av. Diss. | Diss./SD | Contrib.% | Cum.% |
| *Ampelisca diadema* | 0 | 2.54 | 2.56 | 4.24 | 3.59 | 21.63 |
| *Aponuphis bilineata* | 0 | 2.22 | 2.21 | 9.50 | 3.10 | 31.30 |
| *Euclymene oerstedii* | 0 | 2.63 | 2.63 | 5.97 | 3.69 | 18.05 |
| *Jasmineira caudata* | 0 | 2.55 | 2.42 | 1.28 | 3.40 | 25.03 |
| *Jasmineira elegans* | 3.02 | 1.09 | 2.13 | 1.73 | 2.99 | 37.37 |
| *Kurtiella bidentata* | 0.61 | 3.43 | 2.74 | 3.42 | 3.84 | 14.35 |
| *Melinna palmata* | 0 | 2.26 | 2.26 | 4.19 | 3.17 | 28.21 |
| *Prionospio* sp | 0 | 2.24 | 2.19 | 1.28 | 3.08 | 34.38 |
| *Spiophanes bombyx* | 0 | 3.81 | 3.75 | 3.65 | 5.27 | 5.27 |
| *Tubificoides swirencoides* | 3.86 | 0 | 3.74 | 3.56 | 5.24 | 10.51 |
| **Groups LR, DS** | | | | | | |
| Average dissimilarity = 63.39 | | | | | | |
| **Species** | **LR** | **DS** |  |  |  |  |
|  | Av. Abund. | Av. Abund. | Av. Diss. | Diss./SD | Contrib.% | Cum.% |
| *Apseudopsis latreillii* | 2.36 | 0 | 2.19 | 2.81 | 3.45 | 23.76 |
| *Capitella capitata* | 3.92 | 1.95 | 2.05 | 1.06 | 3.24 | 27.00 |
| *Chrysallida* sp | 2.51 | 0 | 2.33 | 3.70 | 3.68 | 9.69 |
| *Cossura soyeri* | 1.79 | 0 | 1.74 | 1.26 | 2.74 | 32.60 |
| *Leptocheirus pilosus* | 0 | 2.46 | 2.23 | 8.60 | 3.52 | 16.80 |
| *Mytilus galloprovincialis* | 3.63 | 1.21 | 2.22 | 1.07 | 3.51 | 20.31 |
| *Prionospio* sp | 2.34 | 0 | 2.28 | 1.25 | 3.59 | 13.28 |
| *Sternaspis scutata* | 1.91 | 3.97 | 1.69 | 1.40 | 2.67 | 35.27 |
| *Tritia nitida* | 2.52 | 0.61 | 1.81 | 1.64 | 2.86 | 29.86 |
| *Tubificoides swirencoides* | 5.99 | 2.25 | 3.81 | 1.26 | 6.01 | 6.01 |
| **Groups LR, API** | | | | | | |
| Average dissimilarity = 69.56 | | | | | | |
| **Species** | **LR** | **API** |  |  |  |  |
|  | Av. Abund. | Av. Abund. | Av. Diss. | Diss./SD | Contrib.% | Cum.% |
| *Abra alba* | 2.91 | 0.71 | 1.77 | 1.93 | 2.54 | 32.13 |
| *Ampelisca diadema* | 0 | 2.54 | 2.03 | 4.72 | 2.92 | 21.18 |
| *Aponuphis bilineata* | 0 | 2.22 | 1.76 | 12.58 | 2.53 | 34.66 |
| *Apseudopsis latreillii* | 2.36 | 0 | 1.88 | 3.39 | 2.71 | 29.59 |
| *Chrysallida* sp | 2.51 | 0 | 2.01 | 5.32 | 2.89 | 24.07 |
| *Euclymene oerstedii* | 0 | 2.63 | 2.10 | 6.99 | 3.01 | 18.26 |
| *Jasmineira caudata* | 0 | 2.55 | 1.95 | 1.29 | 2.80 | 26.88 |
| *Mytilus galloprovincialis* | 3.63 | 0 | 2.78 | 1.99 | 3.99 | 15.25 |
| *Spiophanes bombyx* | 0 | 3.81 | 3.00 | 3.68 | 4.31 | 11.25 |
| *Tubificoides swirencoides* | 5.99 | 0 | 4.83 | 3.12 | 6.94 | 6.94 |
| **Groups DS, API** | | | | | | |
| Average dissimilarity = 71.38 | | | | | | |
| **Species** | **DS** | **API** |  |  |  |  |
|  | Av. Abund. | Av. Abund. | Av. Diss. | Diss./SD | Contrib.% | Cum.% |
| *Aponuphis bilineata* | 0 | 2.22 | 2.25 | 4.64 | 3.15 | 33.05 |
| *Euclymene oerstedii* | 0 | 2.63 | 2.69 | 3.91 | 3.76 | 19.46 |
| *Jasmineira caudata* | 0 | 2.55 | 2.47 | 1.23 | 3.46 | 26.45 |
| *Kurtiella bidentata* | 0 | 3.43 | 3.47 | 4.48 | 4.86 | 15.69 |
| *Leptocheirus pilosus* | 2.46 | 0 | 2.46 | 7.59 | 3.44 | 29.89 |
| *Lumbrineris latreilli* | 0 | 2.52 | 2.53 | 3.97 | 3.54 | 23.00 |
| *Prionospio cirrifera* | 0 | 2.04 | 2.21 | 1.00 | 3.10 | 39.28 |
| *Prionospio* sp | 0 | 2.24 | 2.24 | 1.23 | 3.13 | 36.18 |
| *Spiophanes bombyx* | 0 | 3.81 | 3.83 | 3.02 | 5.36 | 10.83 |
| *Sternaspis scutata* | 3.97 | 0 | 3.90 | 11.78 | 5.47 | 5.47 |

Av. Abund. = Average Abundance; Av. Diss. = Average Dissimilarity; Diss. = Dissimilarity; SD = Standard Deviation; Contrib. = Contribution; Cum. = Cumulative contribution.

**Table S10. RELATE test showing correlations between meiofauna and macrofauna.** Significant correlations are in bold.

|  |  |  | **Macrobenthic taxonomic level** | **Rho** | **Significance level [%]** |
| --- | --- | --- | --- | --- | --- |
| **With Port Station** | Meiofauna | vs | Species | 0.269 | **2.78** |
|  | Meiofauna | vs | Family | 0.267 | **3.45** |
|  | Meiofauna | vs | Class | 0.008 | 43.77 |
|  | Meiofauna | vs | Phylum | 0.104 | 23.07 |
|  |  |  |  |  |  |
| **Without Port Station** | Meiofauna | vs | Species | 0.621 | **0.04** |
|  | Meiofauna | vs | Family | 0.554 | **0.07** |
|  | Meiofauna | vs | Class | 0.416 | **0.36** |
|  | Meiofauna | vs | Phylum | 0.388 | **1.12** |
| PRIMER's RELATE routine; number of permutations = 9999; alpha = 0.05 | | | | | |
