# Supplementary figures and images for: Multi-benthic size approach to unveil different environmental conditions in a Mediterranean harbor area (Ancona, Adriatic Sea, Italy)
Source: PeerJ. 2023 Jun 28;11:e15541. doi: 10.7717/peerj.15541 (PMC10314744; doi:10.7717/peerj.15541)

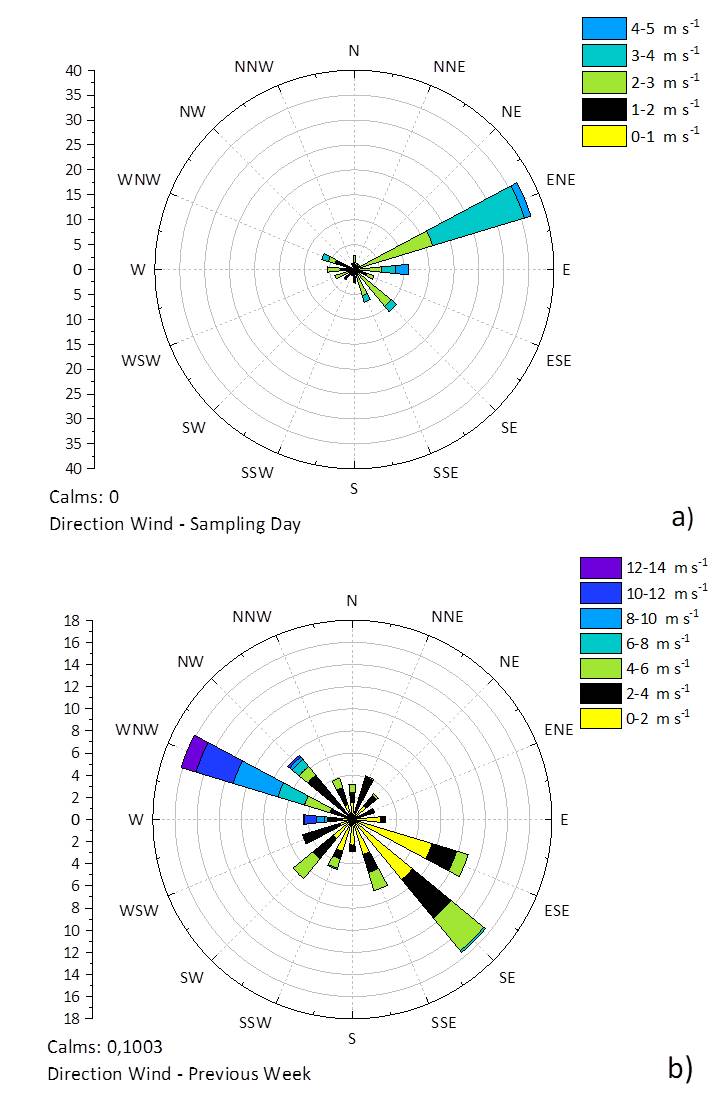

Supplement: Supplemental Information 2 — Data retrieved from Ancona Harbor station (Latitude 43°36.579 N, Longitude 13°28.912 E) of the ISPRA (Italian Institute for Environmental Protection and Research) national tide gauge network (Rete Mareografica Nazionale). [file peerj-11-15541-s002.jpg]

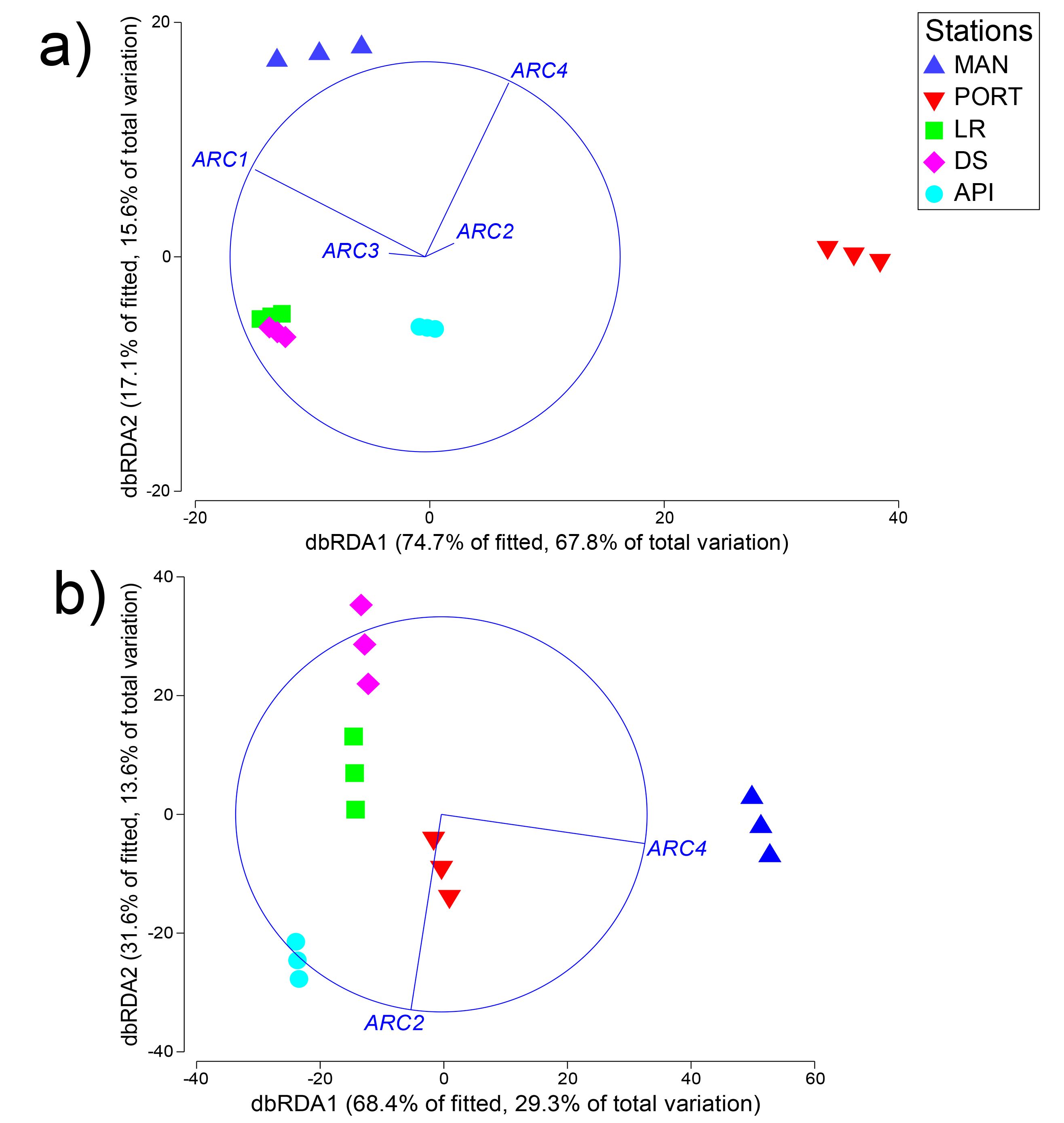

Supplement: Supplemental Information 3 — Distance-based Redundancy Analysis (dbRDA) graphs on (A) meiofauna and (B) macrofauna compositions, showing the sampling station distribution according to selected environmental variables. The first two axes explain (A) the 91.8% of the variability and (B) the 100% of the variability. Variables are coded after Varimax Rotated PCA Axis as follow: ARCx = Pollutant variables associated to the rotated axis (x indicates the number of the axis). For a complete list of the variables refers to the text. [file peerj-11-15541-s003.jpg]
